# Nerve growth factor gene therapy improves bone marrow sensory innervation and nociceptor-mediated stem cell release in a mouse model of type 1 diabetes with limb ischaemia

**DOI:** 10.1007/s00125-019-4860-y

**Published:** 2019-04-24

**Authors:** Zexu Dang, Elisa Avolio, Ambra Albertario, Graciela B. Sala-Newby, Anita C. Thomas, Nianhong Wang, Costanza Emanueli, Paolo Madeddu

**Affiliations:** 1 Experimental Cardiovascular Medicine, Faculty of Translational Health Sciences, Bristol Medical School, University of Bristol, Upper Maudlin Street, Bristol, BS2 8HW UK; 2 Department of Rehabilitation Medicine, Huashan Hospital, Fudan University, Pudong, Shanghai, China; 3 National Heart and Lung Institute, Imperial College London, London, UK

**Keywords:** Bone marrow, Bone marrow stem cells, Gene therapy, Nerve growth factor, Nociceptor, PC12 cells, Peripheral ischaemia, Sensory neuropathy, Substance P, Type 1 diabetes

## Abstract

**Aims/hypothesis:**

Sensory neuropathy is common in people with diabetes; neuropathy can also affect the bone marrow of individuals with type 2 diabetes. However, no information exists on the state of bone marrow sensory innervation in type 1 diabetes. Sensory neurons are trophically dependent on nerve growth factor (NGF) for their survival. The aim of this investigation was twofold: (1) to determine if sensory neuropathy affects the bone marrow in a mouse model of type 1 diabetes, with consequences for stem cell liberation after tissue injury; and (2) to verify if a single systemic injection of the *NGF* gene exerts long-term beneficial effects on these phenomena.

**Methods:**

A mouse model of type 1 diabetes was generated in CD1 mice by administration of streptozotocin; vehicle was administered to non-diabetic control animals. Diabetic animals were randomised to receive systemic gene therapy with either human *NGF* or β-galactosidase. After 13 weeks, limb ischaemia was induced in both groups to study the recovery post injury. When the animals were killed, samples of tissue and peripheral blood were taken to assess stem cell mobilisation and homing, levels of substance P and muscle vascularisation. An in vitro cellular model was adopted to verify signalling downstream to human *NGF* and related neurotrophic or pro-apoptotic effects. Normally distributed variables were compared between groups using the unpaired Student’s *t* test and non-normally distributed variables were assessed by the Wilcoxon–Mann–Whitney test. The Fisher’s exact test was employed for categorical variables.

**Results:**

Immunohistochemistry indicated a 3.3-fold reduction in the number of substance P-positive nociceptive fibres in the bone marrow of type 1 diabetic mice (*p* < 0.001 vs non-diabetic). Moreover, diabetes abrogated the creation of a neurokinin gradient which, in non-diabetic mice, favoured the mobilisation and homing of bone-marrow-derived stem cells expressing the substance P receptor neurokinin 1 receptor (NK1R). Pre-emptive gene therapy with *NGF* prevented bone marrow denervation, contrasting with the inhibitory effect of diabetes on the mobilisation of NK1R-expressing stem cells, and restored blood flow recovery from limb ischaemia. In vitro hNGF induced neurite outgrowth and exerted anti-apoptotic actions on rat PC12 cells exposed to high glucose via activation of the canonical neurotrophic tyrosine kinase receptor type 1 (TrkA) signalling pathway.

**Conclusions/interpretation:**

This study shows, for the first time, the occurrence of sensory neuropathy in the bone marrow of type 1 diabetic mice, which translates into an altered modulation of substance P and depressed release of substance P-responsive stem cells following ischaemia. *NGF* therapy improves bone marrow sensory innervation, with benefits for healing on the occurrence of peripheral ischaemia. Nociceptors may represent a new target for the treatment of ischaemic complications in diabetes.

**Electronic supplementary material:**

The online version of this article (10.1007/s00125-019-4860-y) contains peer-reviewed but unedited supplementary material, which is available to authorised users.

## Introduction

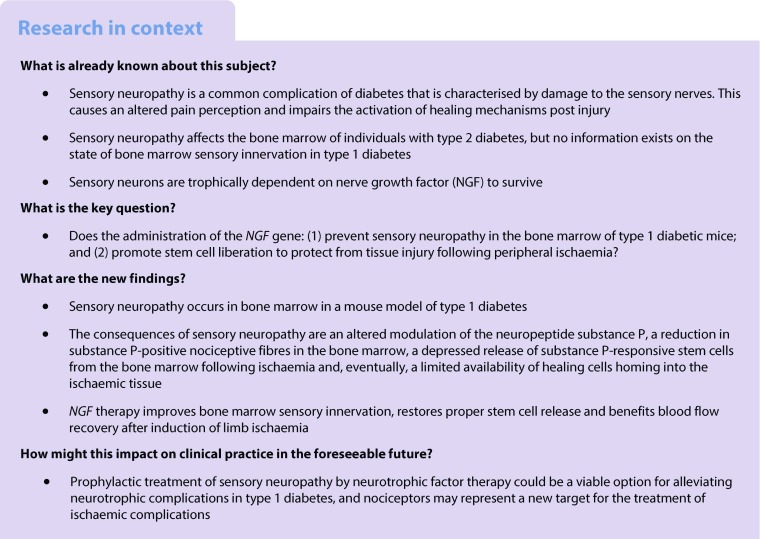



Diabetes mellitus is a great challenge for the healthcare system, accounting for ~6% of mortality in industrialised countries [1]. As many as 70% of people with diabetes are estimated to have some form of neuropathy, which often overlaps with, and worsens, the consequences of diabetic vascular disease [2, 3]. Sensory neuropathy is a typical form of peripheral neuropathy, characterised by an altered perception of noxious stimuli and ischaemic pain [4–6]. Impaired nociception facilitates the insurgence of foot ulcers caused by pressure or traumas [4] and also abrogates warning symptoms during a heart attack [7].

Our group has recently reported that sensory neuropathy can occur in the bone marrow of mice and individuals with long-term type 2 diabetes. Importantly, sensory fibre rarefaction was associated with an impairment in the neurokinin gradient that triggers the release of bone-marrow-derived pro-angiogenic cells expressing the substance P neurokinin 1 receptor (NK1R) [8]. Nevertheless, no investigation has yet been conducted to ascertain the presence and consequences of bone marrow neuropathy in type 1 diabetes. This is an important issue as neuropathic complications are equally prevalent and therapeutically challenging in both types of diabetes [9].

Experimental and clinical evidence suggests that a deficit in neurotrophic factor signalling contributes to the pathogenesis of diabetic sensory [10, 11] and autonomic neuropathy [12]. Therefore, supplementation with neurotrophic factors, such as nerve growth factor (NGF) and glial-cell-line-derived neurotrophic factor (GDNF), could be a viable option to treat neuropathic complications, with the only caveat that hyperalgesia has been reported as an occasional side effect of NGF in clinical trials [13, 14].

**Fig. 1:**
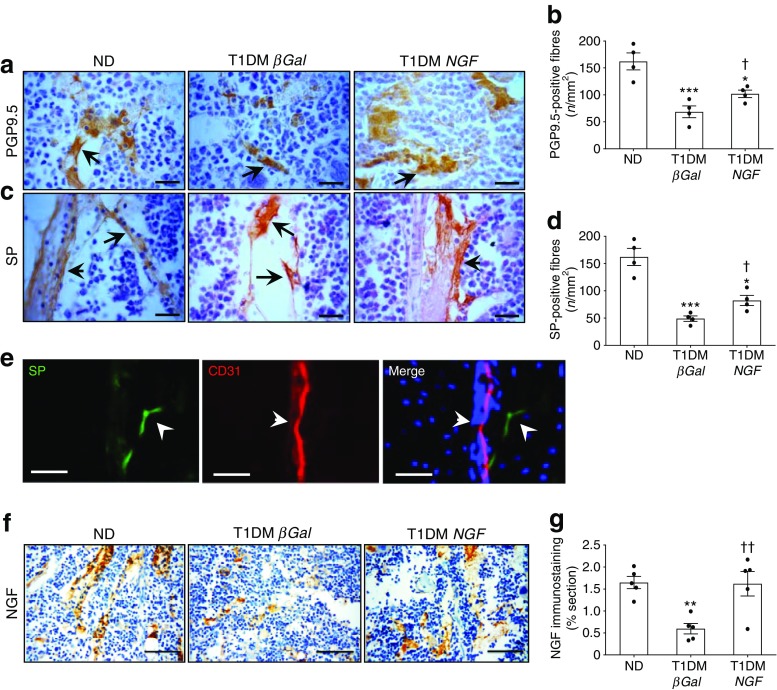
Gene therapy with *NGF* prevents bone marrow neuropathy. Immunohistochemical studies were carried out to compare the nerve fibre density in the bone marrow of non-diabetic and type 1 diabetic mice. The latter were randomised to receive Ad.h*NGF* or Ad.*βGal*. (**a**–**d**) Representative micrographs and bar graphs showing the density of neuronal fibres expressing the pan-neuronal marker PGP9.5 (**a**, **b**) and nociceptive fibres positive for substance P (**c**, **d**) (scale bar, 20 μm). Arrows point to positive fibres. (**e**) Representative immunofluorescence microscopy images identify substance P-containing sensory terminals, which were often associated with CD31-positive vascular structures (arrows) (scale bar, 50 μm). (**f**, **g**) Representative micrographs and bar graphs showing the density of NGF-positive neuronal fibres in bone marrow (scale bar, 50 μm). *n* = 4 per group (**b**, **d**); *n* = 5 per group (**g**). Data are expressed as means ± SEM. ^*^p < 0.05, ^**^p < 0.01 and ^***^p < 0.001 vs non-diabetic animals; ^†^*p* < 0.05 and ^††^*p* < 0.01 vs type 1 diabetic mice with Ad.*βGal*. ND, non-diabetic; SP, substance P; T1DM, type 1 diabetic

We have previously shown that NGF supplementation has pleiotropic therapeutic activities on nerves and vessels via a trophic signalling pathway comprising the neurotrophic tyrosine kinase receptor type 1 (TrkA), Akt and nitric oxide synthase (NOS), which overrides proNGF-induced activation of the pro-apoptotic p75 neurotrophin receptor (p75^NTR^) [15, 16] (this dual opposing mechanism is schematically illustrated in electronic supplementary material [ESM] Fig. 1). We were also the first to demonstrate that adenovirus (Ad)-mediated *NGF* cardiac gene therapy induced the mobilisation and homing of pro-angiogenic bone-marrow-derived progenitor cells into ischaemic tissues [17, 18].

The aim of the present investigation was twofold: to determine the occurrence of sensory neuropathy in the bone marrow in a mouse model of type 1 diabetes (early stage) and to verify whether systemic *NGF* gene therapy can prevent such a bone marrow pathology and the consequent defect in progenitor cell mobilisation following limb ischaemia.

## Methods

An extended, detailed version of the methods is available as ESM. All manufacturers and suppliers are reported in the ESM. In in vitro experiments, each biological sample was assayed using 2–4 technical replicates, and the values average was used for statistical analysis. Randomisation did not apply to in vitro studies and experimenter blinding was not carried out.

### In vivo model of type 1 diabetes, limb ischaemia and gene therapy

The experimental protocol is summarised in ESM Fig. 2.

Type 1 diabetes was induced in male 7-week-old CD1 mice by intraperitoneal injection of streptozotocin (STZ). CD1 is a multipurpose mouse model, using an outbred and genetic stable strain, and is available from Charles River for biomedical research; the full name is Crl:CD1(ICR). Mice with STZ-induced diabetes develop a form of neuropathy like that seen in individuals with type 1 diabetes. Age-matched male CD1 mice injected with STZ vehicle were used as non-diabetic control animals. At 2 weeks after the first STZ injection, only mice that had developed diabetes were included in the study, underwent randomisation to two experimental groups and were injected with either adenovirus carrying the human *NGF* gene (Ad.h*NGF*) or adenovirus carrying the β-galactosidase gene (Ad.*βGal*; *βGal* is also known as *Glb1*) via the tail vein (total dose of 1.5 × 10^9^ viral particles in 100 μl). Age-matched non-diabetic mice were used for reference samples collected after 16 weeks.

In a second set of experiments, unilateral limb ischaemia was induced in mice 13 weeks after gene delivery. Animals were killed at 0, 1, 3, 7, 14 and 21 days after limb ischaemia.

An additional set of CD1 mice were given Ad.h*NGF* or Ad.*βGal* and plasma was collected 3 days later for assessment of hNGF overexpression.

### Immunohistochemistry and immunofluorescence microscopy

Bone marrow neuronal fibres, NGF, V5-tag, substance P and phospho-ribosomal protein S6 (phospho-rpS6) were evaluated in bone marrow sections or dorsal root ganglia. Capillary and arteriole densities were quantified in muscle sections. The specificity of the anti-NGF antibody was confirmed with a competitive assay using an NGF peptide.

### ELISA

ELISAs for hNGF, pro-hNGF and substance P were performed on plasma, bone marrow or ischaemic adductor muscles.

### Rat fibroblast transduction and collection of conditioned media

Primary rat fibroblasts were transduced with either Ad.h*NGF* or Ad.*βGal*. Cells not transduced served as control (non-virus). After 48 h, the conditioned medium was collected and used for subsequent studies with rat PC12 cells (a neuronal cell line derived from a pheochromocytoma of the rat adrenal medulla).

### Biological assays on PC12 cells exposed to the conditioned media from NGF-transduced fibroblasts

To test the biological effects of hNGF, PC12 cells were pre-conditioned for 48 h with basal- or high-glucose medium (5 mmol/l and 30 mmol/l, respectively) and exposed to conditioned medium from fibroblasts previously transduced with either Ad.h*NGF* or Ad.*βGal*. Whole cell protein extracts were collected for signalling studies by western blotting. Apoptosis and neuronal differentiation experiments were performed.

### Flow cytometry on peripheral blood progenitor cells

Analyses of progenitor cell phenotype in peripheral blood, bone marrow and adductor muscles of mice were conducted using multicolour flow cytometry.

### Bone marrow cell migration

To verify the effect of type 1 diabetes on the sensitivity of bone marrow cells to substance P, in vitro migration assays of freshly isolated bone marrow cells to substance P were performed.

### Ethics approval and consent to participate

Experiments involving live animals were performed in accordance with the Guide for the Care and Use of Laboratory Animals (Institute of Laboratory Animal Resources, 1996) and with approval from the British Home Office and the University of Bristol.

### Statistical analysis

The D’Agostino and Pearson omnibus normality test was applied to check for normal distribution of data. Normally distributed continuous variables were expressed as mean ± SEM and compared between groups using the unpaired Student’s *t* test. When continuous variables did not follow a normal distribution, values were expressed as median (interquartile range [IQR]) and compared using the Wilcoxon–Mann–Whitney test. Categorical variables were compared using the *χ*^2^ or Fisher’s exact test. All reported *p* values are two sided. A *p* value <0.05 was considered statistically significant.

## Results

### Rarefaction of nociceptive innervation in bone marrow from type 1 diabetic mice

We first compared the abundance of nerve fibres in the bone marrow of mice with early-stage type 1 diabetes and non-diabetic control animals using immunohistochemical techniques described previously [19, 20]. Figure 1a, c shows fibres that express the pan-neuronal markers PGP9.5 and substance P in the central part of the bone cavity and the periosteum. Morphometric analyses showed that diabetes induces a marked reduction in the density of both PGP9.5-positive fibres (Fig. 1b, *p* < 0.001 vs non-diabetic animals) and substance P-containing sensory terminals (Fig. 1d, *p* < 0.001 vs non-diabetic animals).

Using immunofluorescence microscopy, we recognised that substance P-containing sensory terminals were often associated with CD31-positive vascular structures (Fig. 1e). In addition, substance P-positive sensory fibres in the bone marrow express the neurotrophic factor receptors TrkA, p75^NTR^ and receptor for GDNF-family ligands (RET) (ESM Fig. 3). Specificity of the substance P immunofluorescent signal was confirmed in spinal cord sections, where substance P was found typically localised in dorsal root ganglia (ESM Fig. 4).

### Systemic *NGF* gene therapy prevents bone marrow neuropathy

NGF reportedly produces a robust regeneration of peripheral nociceptive axons [21]. To investigate whether *NGF* gene therapy prevents bone marrow sensory neuropathy, we delivered an Ad.h*NGF* construct, described previously [18, 22], to mice intravenously, 2 weeks after induction of diabetes by STZ. After 16 weeks, bone marrow and spinal cord were collected to perform immunohistochemical studies. Interestingly, *NGF* gene therapy prevented the reductive effect of diabetes on PGP9.5- and substance P-positive fibre density (Fig. 1a–d, *p* < 0.05 vs *βGal* mice for both comparisons). Also, we found that NGF protein expression is downregulated in bone marrow nerves of diabetic mice compared with non-diabetic mice (Fig. 1f, g, *p* < 0.01). The co-expression of NGF and PGP9.5 by neuronal fibres was verified in consecutive bone marrow sections (ESM Fig. 5). The h*NGF* gene transfer was able to restore normal NGF levels in bone marrow neuronal fibres (Fig. 1f, g, *p* < 0.01 vs diabetic mice given *βGal*]). Together, these results indicate that *NGF* gene therapy can prevent neuropathy in diabetic bone marrow.

**Fig. 2:**
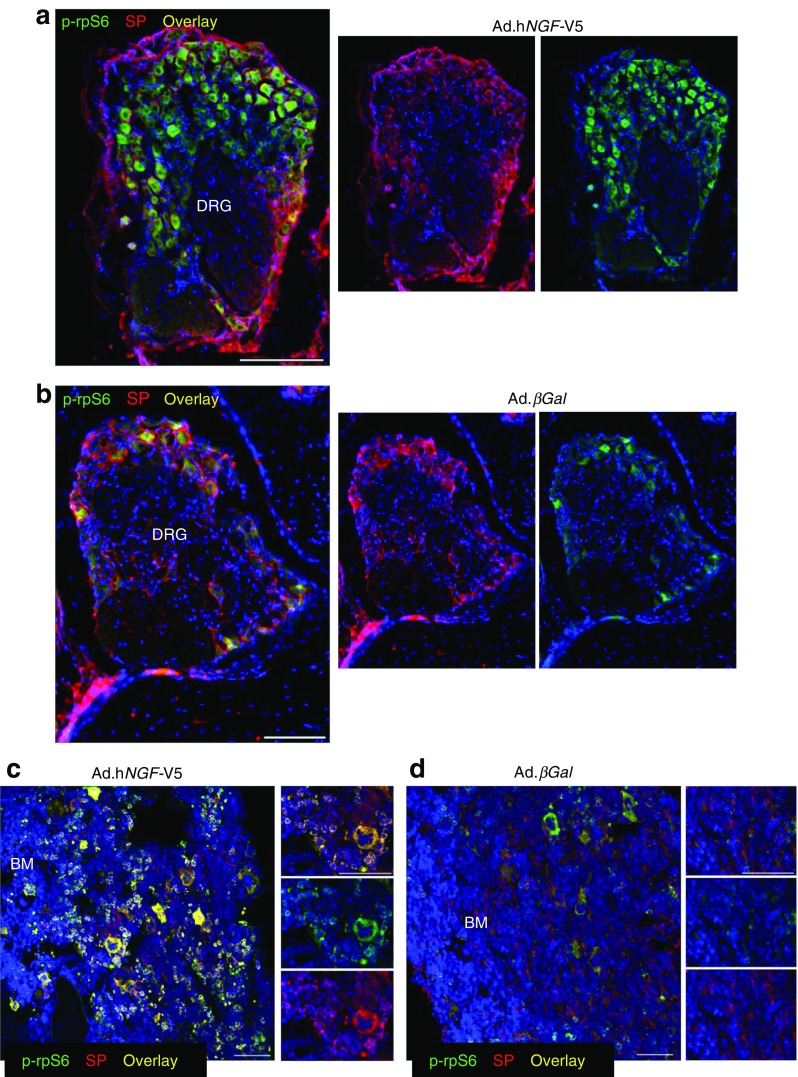
Gene therapy with *NGF* activates TrkA signalling in sensory neurons and bone marrow cells of diabetic mice. (**a**, **b**) Representative immunofluorescence images showing that, 16 weeks post gene transfer, a higher number of substance P-positive sensory neuron bodies display phosphorylation of rpS6 in the DRG of diabetic mice receiving V5-tagged Ad.h*NGF* (**a**) compared with diabetic mice receiving Ad.*βGal* (**b**). Scale bar, 100 μm. (**c**, **d**) Representative immunofluorescence images showing that, in bone marrow of h*NGF*-treated mice, a higher number of cells are characterised by phosphorylation of rpS6 (**c**) compared with control mice receiving *βGal* (**d**). Scale bar, 50 μm. In all images, phospho-rpS6 is shown in green, substance P in red. DAPI, in blue, identifies nuclei. *n* = 3 mice, Ad.h*NGF* and *n* = 3 mice, Ad.*βGal*. BM, bone marrow; SP, substance P

We then confirmed that NGF overexpression activated the TrkA signalling in the sensory neurons, and with this aim, we analysed the cell bodies of sensory neurons located in the dorsal root ganglia (DRG). At 16 weeks post gene therapy, we observed that a higher number of substance P-positive neurons showed phosphorylation of ribosomal protein S6 (P-rpS6) in diabetic animals receiving h*NGF* compared with diabetic mice receiving *βGal* (Fig. 2a, b). Also, we observed a higher number of cells co-expressing substance P and P-rpS6 in the bone marrow of *NGF*-treated mice (Fig. 2c, d). Phosphorylation of rpS6 is associated with cell growth and survival, suggesting therapy with *NGF* confers protective effects on multiple cell types and may contribute to preservation of the general homeostasis of the bone marrow environment.

**Fig. 3:**
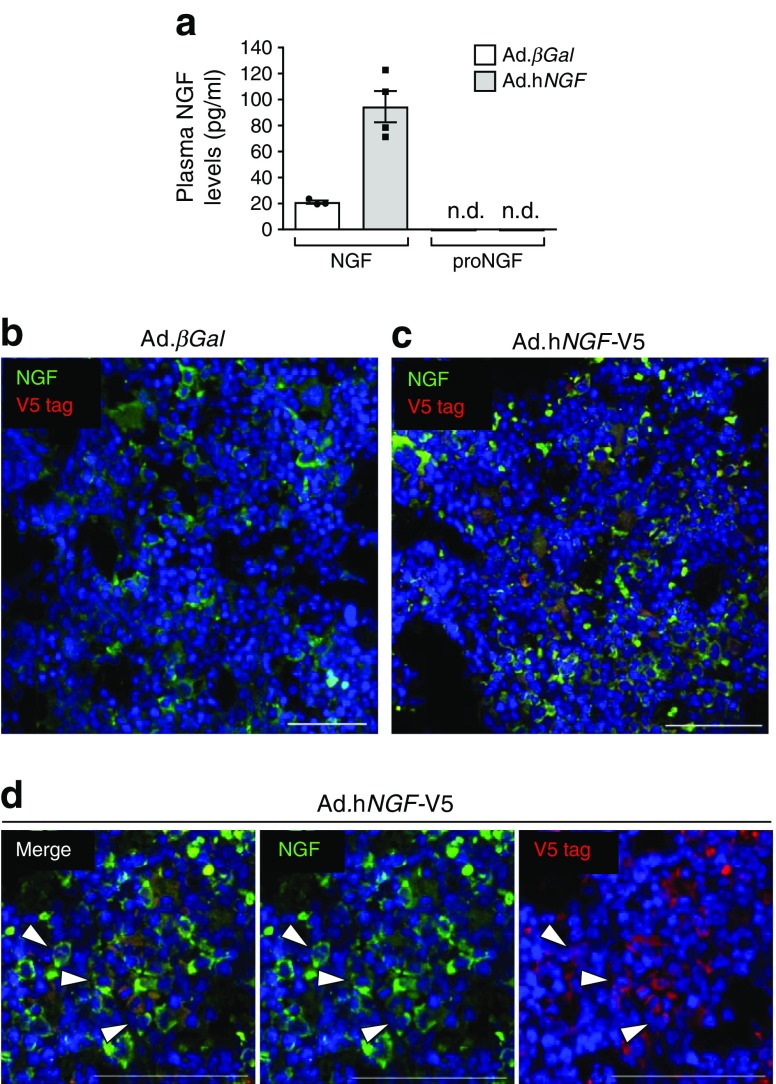
Expression of recombinant hNGF in mouse plasma and bone marrow. (**a**) The expression of NGF and proNGF was measured by ELISA assay in mouse plasma 3 days post gene transfer (*n* = 4 Ad.h*NGF* and *n* = 3 Ad.*βGal*). Data are expressed as means ± SEM. (**b**–**d**) The recombinant V5-tagged hNGF was detected by immunofluorescent staining in bone marrow cells of mice receiving gene therapy, at the end of the experimental procedure (16 weeks post gene transfer). Animals given Ad.*βGal* served as a negative control for the V5 staining. V5 is shown in red, NGF in green, and blue (DAPI) shows nuclei. Arrows point to some examples of double-positive cells. Scale bar, 50 μm. n.d., not detected

The overexpression of NGF was confirmed by ELISA on plasma samples 3 days post gene transfer (average of 94 ± 12 pg/ml in the Ad.h*NGF* group vs 21 ± 1 pg/ml in the Ad.*βGal* group) (Fig. 3a). Importantly, no circulating proNGF was detected. Moreover, immunohistochemical analysis showed bone marrow cells expressing the V5-tagged hNGF 16 weeks after gene therapy (Fig. 3b–d). Specificity of the anti-NGF antibody was confirmed with a competitive assay in which the antibody was neutralised with the immunising NGF peptide (ESM Fig. 6).

### h*NGF*-transduced cells exert paracrine-protective effects on PC12 cells

**Fig. 4:**
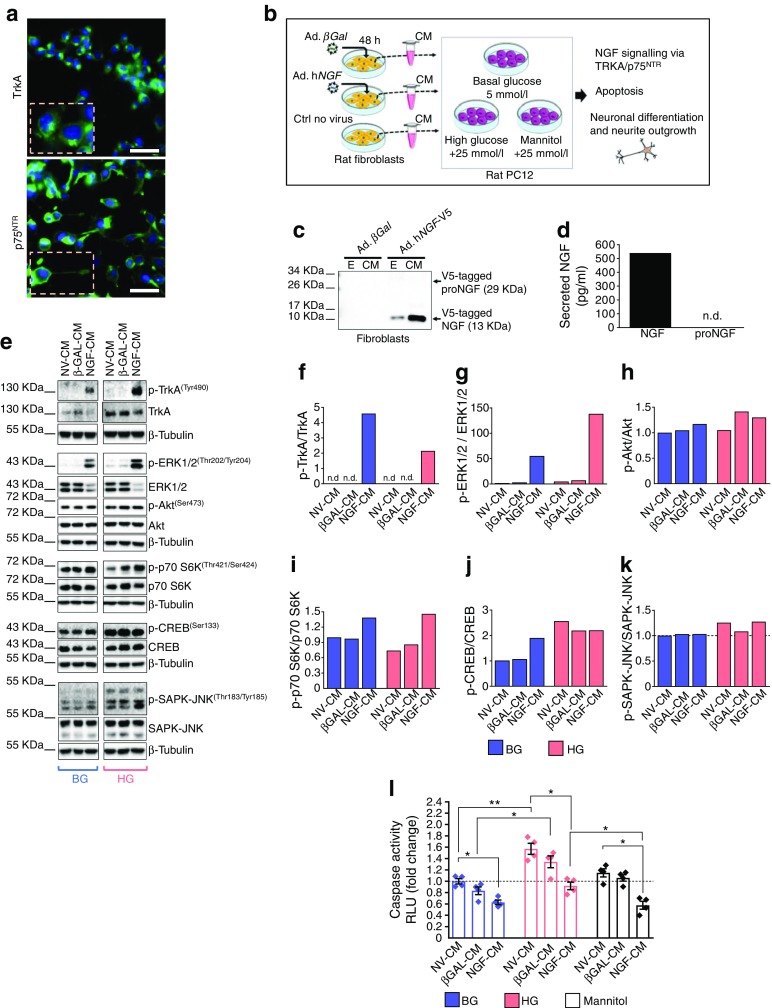
hNGF exerts neuroprotective effects in PC12 cells in vitro. (**a**) PC12 cells express the NGF receptors TrkA and p75^NTR^ (green). DAPI identifies nuclei (blue). Scale bar, 50 μm. (**b**) Schematic showing the experimental model. Rat fibroblasts were transduced with Ad.h*NGF* or Ad.*βGal*; non-transduced cells served as the control. Conditioned medium from fibroblasts was collected after 48 h and used to mimic the paracrine action of circulating NGF on target PC12 cells in a basal- or high-glucose environment, or with mannitol as osmotic control. Endpoints of the experiments were NGF intracellular signalling, apoptosis assay and neural differentiation. (**c**) V5-tagged hNGF was detected by western blotting in fibroblast total cellular extracts and conditioned medium. Only the mature NGF was detected, indicating that preproNGF was successfully cleaved to the mature form. (**d**) ELISA detected 540 pg/ml mature NGF in the fibroblast-conditioned medium used for the experiments. No proNGF was detected. (**e**) Blots for phospho-proteins and corresponding non-phosphorylated forms associated with pro-survival TrkA and pro-apoptotic p75^NTR^ signalling. (**f**–**k**) Graphs showing blot densitometry (*n* = 1). In (**g**–**k**), values are expressed as fold change of the basal glucose non-virus-conditioned medium. (**l**) Bar graph showing caspase 3/7 activity in PC12 exposed to either basal or high glucose or mannitol, for 48 h, in the presence of fibroblast-conditioned medium. Caspase activity, measured as relative luminescence units, is expressed as fold change of the basal glucose non-virus-conditioned-medium group (*n* = 4 per group). Data are expressed as means ± SEM. ^*^p < 0.05 and ^**^p < 0.01 between groups, as indicated. BG, basal glucose; CM, conditioned medium; Ctrl, control; E, total cellular extract; HG, high glucose; n.d., not detected; NV, non-virus; RLU, relative luminescence units

We next performed in vitro assays on PC12 cells and verified that these cells express the NGF receptors TrkA and p75^NTR^ (Fig. 4a). Rat fibroblasts were transduced with either Ad.h*NGF* or Ad.*βGal* and their conditioned medium was used for conditioning PC12 cells under basal- or high-glucose conditions (Fig. 4b). Results of western blotting confirmed the presence of hNGF in fibroblast-conditioned media (Fig. 4c), with immunoblotting with an anti-V5 tag antibody identifying a band of ~13 kDa, corresponding to the mature hNGF in the conditioned medium and cell extracts. ProNGF was not detected, indicating that the recombinant protein is cleaved to its mature form as physiologically expected for the endogenous NGF. These results were further confirmed using ELISAs to identify the presence of NGF in the cell-conditioned medium (540 pg/ml). In contrast, proNGF was not detected by the assay (Fig. 4d).

Western blot analyses showed that the conditioned medium of hNGF-transduced fibroblasts activated TrkA under conditions of either basal or high glucose, as indicated by the phosphorylation of tyrosine 490 (Fig. 4e, f). This event triggers the classic pro-survival signalling via TrkA in PC12 cells, as suggested by increased phosphorylation of extracellular signal-regulated kinases 1/2 (ERK1/2), S6K and cAMP response element binding protein (CREB) compared with PC12 cells exposed to non-virus- and βGAL-conditioned medium; Akt phosphorylation was not relevantly modified at the time point assessed (Fig. 4e, g–j). We observed a slight increase in stress-activated protein kinase (SAPK)-Jun N-terminal kinase (JNK) phosphorylation in all groups exposed to high-glucose conditions (Fig. 4e). SAPK-JNK phosphorylation is involved in p75^NTR^ pro-apoptotic signalling [23]. However, we could not find any change in SAPK-JNK phosphorylation levels following the exposure of PC12 to NGF-conditioned medium (Fig. 4k) We also performed a caspase 3/7 assay in PC12 cells incubated with the fibroblast-conditioned medium for 48 h, with either basal or high glucose levels, or mannitol as control (Fig. 4l). We found that high glucose, but not treatment with the osmotic control mannitol, induced apoptosis in PC12 cells in the non-virus- and βGAL-conditioned-medium groups (*p* < 0.01 and *p* < 0.05 vs corresponding basal glucose groups). Importantly, the conditioned medium of hNGF-transduced fibroblasts was able to prevent cell apoptosis in PC12 cells exposed to high glucose (*p* < 0.05, *NGF* vs non-virus). It also reduced apoptosis in PC12 cells exposed to basal glucose or mannitol (*p* < 0.05 vs non-virus-conditioned medium, for both comparisons).

**Fig. 5:**
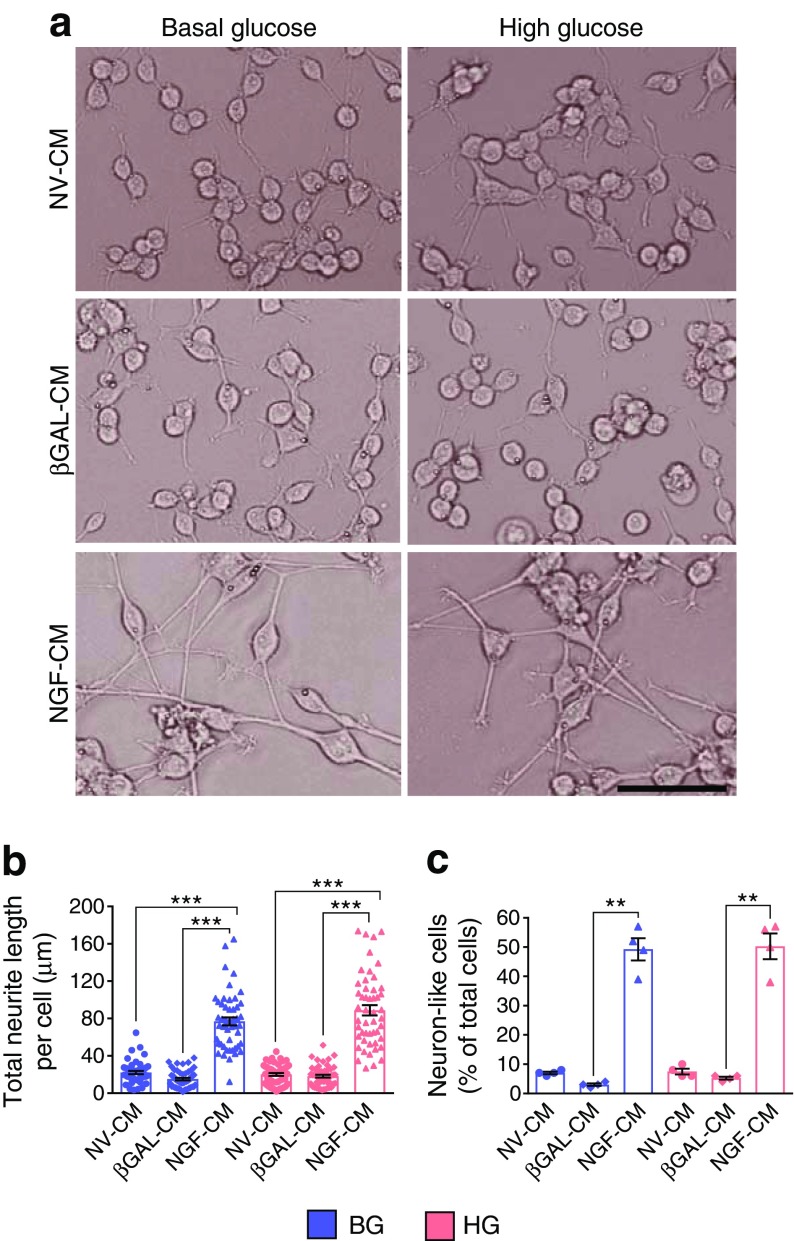
hNGF induces neuronal differentiation of PC12 cells in vitro. (**a**) Bright-field images showing that PC12 cells differentiate into neuron-like cells in the presence of hNGF for 3 days, in either basal- or high-glucose environments (for the schematic of the experiment, please see Fig. 4b). Scale bar, 50 μm. (**b**) Graph showing the total neurite length per cell (*n* = 50 cells per group assessed in four different imaging fields). (**c**) Graph showing the percentage of neuron-like cells, defined as cells presenting at least one axon longer than the cell body (*n* = 4 different imaging fields per group, for a total of *n* = 250–300 cells assessed per group). Data are expressed as means ± SEM. ^**^p < 0.01 and ^***^p < 0.001 between groups, as indicated. Blue bars/symbols, basal glucose; pink bars/symbols, high glucose. BG, basal glucose; CM, conditioned medium; HG, high glucose; NV, non-virus

Finally, we tested the biological activity of the conditioned medium of h*NGF*-transduced fibroblasts in a neural differentiation assay. As shown in Fig. 5a, this induced neurite outgrowth and differentiation of PC12 cells into neuron-like cells under either basal- or high-glucose conditions. The total neurite length per cell was significantly higher in the NGF-conditioned-medium groups (*p* < 0.0001 vs non-virus-conditioned medium and βGAL-conditioned medium) (Fig. 5b). In addition, the number of neuron-like cells, identified as cells bearing at least one axon longer than the cell body, was significantly higher in the NGF-conditioned-medium groups (*p* < 0.01 vs βGAL-conditioned medium) (Fig. 5c).

Taken together, these in vitro results corroborate the in vivo data, demonstrating that an environmental increase in NGF levels improves the growth and survival of neuronal cells.

### Bone marrow sensory neuropathy is associated with the blunted release of nociceptor-positive progenitor cells following limb ischaemia

**Fig. 6:**
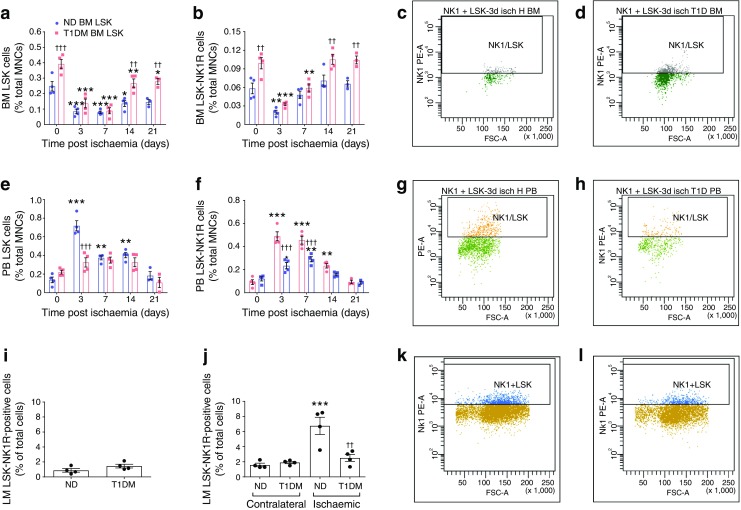
Impaired liberation and homing of nociceptor-expressing cells in type 1 diabetic mice subjected to unilateral limb ischaemia. (**a**–**h**) Flow cytometry analyses showing the abundance of LSK and LSK-NK1R cells in the bone marrow (**a**–**d**) and peripheral blood (**e–h**) of non-diabetic and type 1 diabetic mice before and after induction of limb ischaemia. Data are expressed as percentage of mononuclear cells. Representative images of gating performed on samples of bone marrow: (**c**) non-diabetic and (**d**) type 1 diabetic; and peripheral blood: (**g**) non-diabetic and (**h**) type 1 diabetic, collected at 3 days post limb ischaemia. *n* = 5 mice per group; ^*^p < 0.05, ^**^p < 0.01 and ^***^p < 0.001 vs time 0; ^††^*p* < 0.01 and ^†††^*p* < 0.001 vs non-diabetic animals. (**i**–**l**) Flow cytometry analyses of LSK-NK1R cells in murine muscles. Bar graphs showing the levels of LSK-NK1R cells in normoperfused limb muscles from non-operated mice (**i**), and LSK-NK1R cells in contralateral and ischaemic limb muscles collected 3 days post limb ischaemia from non-diabetic and type 1 diabetic mice (**j**). Typical gates of flow cytometry analyses performed on ischaemic limb muscles from non-diabetic (**k**) and type 1 diabetic (**l**) mice. *n* = 4 per group. ^***^p < 0.001 vs contralateral; ^††^*p* < 0.01 vs non-diabetic. All data are expressed as means ± SEM. BM, bone marrow; FSC-A, forward scatter-area; LM, limb muscle; MNC, mononuclear cell; ND, non-diabetic; PB, peripheral blood; PE-A, phycoerythrin area; T1DM, type 1 diabetic

Having demonstrated that type 1 diabetes induces structural alterations of bone marrow sensory innervation, we next asked if this neuropathology may have functional consequences for the release of haematopoietic progenitor cells following induction of tissue damage. We have previously shown that, in healthy mice, tissue ischaemia induces the liberation of lineage^−^ Sca-1^+^ and c-Kit^+^ (LSK) cells co-expressing the substance P receptor NK1 (LSK-NK1R). This mechanism involves the activation of neuronal circuitry projecting from injured tissues to the bone marrow, which leads to the generation of a chemoattractant gradient of substance P, favouring the movement of LSK-NK1R cells from bone marrow to peripheral tissue [24]. To determine if type 1 diabetes may affect this mechanism, we next performed flow cytometry quantification of LSK-NK1R cells in bone marrow and peripheral blood from non-diabetic and diabetic mice before and after unilateral limb ischaemia. The gating procedure is shown in ESM Fig. 7. As expected, diabetic mice showed retention of LSK and LSK-NK1R cells in bone marrow and blunted release of the same cells into the circulation (*p* < 0.001 vs non-diabetic control animals for both cell types [Fig. 6a–h]). For precise identification of the cells in limb muscle, we first performed flow cytometry analyses of digested adductor muscles harvested from non-ischaemic mice and found that resident LSK-NK1R cells were of low abundance in both non-diabetic and type 1 diabetic mice (~1% of total cells) (Fig. 6i). We next assessed the homing of LSK-NK1R cells in ischaemic and contralateral muscles at 3 days post ischaemia, which corresponds to the peak of cell liberation into the circulation. Low levels of LSK-NK1R cells were detected in contralateral limb muscle with no difference between the non-diabetic and type 1 diabetic groups (Fig. 6j). Ischaemia increased the abundance of LSK-NK1R cells by 4.3-fold in non-diabetic mice (*p* < 0.001 vs contralateral), with this effect being remarkably reduced in diabetic mice (*p* < 0.01 vs non-diabetic mice, Fig. 6j). These data are also illustrated as representative flow cytometry images from ischaemic limb muscle of non-diabetic (Fig. 6k) and type 1 diabetic mice (Fig. 6l).

### Systemic gene therapy with *NGF* improves reparative processes and release of progenitor cells in diabetic mice with limb ischaemia

**Fig. 7:**
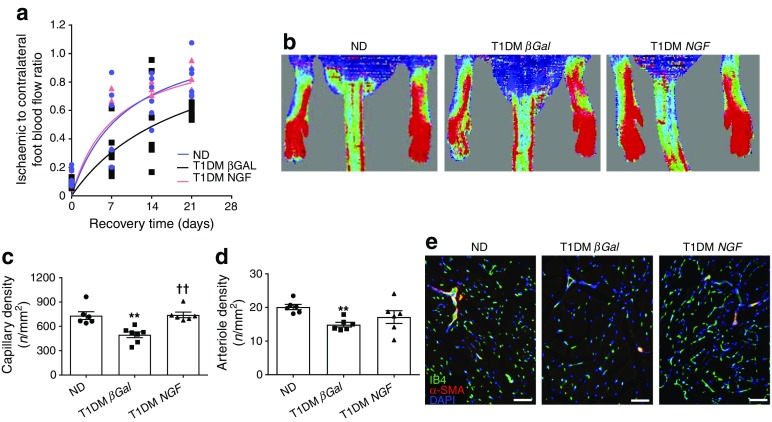
Systemic *NGF* therapy improves recovery from limb ischaemia. (**a**, **b**) Line graph (**a**) and representative laser Doppler images (**b**) of limb muscle reperfusion. (**c**–**e**) Bar graph (**c**, **d**) and representative fluorescent microphotographs (**e**) of capillary and arteriole density in limb muscles of non-diabetic and type 1 diabetic mice treated with Ad.h*NGF* or Ad.*βGal*. (**e**) Capillary endothelial cells are stained with isolectin B4 (green) and arterioles with α-smooth muscle actin (red). Nuclei are stained with DAPI (blue). Scale bar, 50 μm. Bar graphs (**c**, **d**) summarise capillary and arteriole density data. In (**c**) *n* = 6 per non-diabetic group and type 1 diabetic *NGF*; *n* = 7 per type 1 diabetic *βGal*. In (**d**) *n* = 6 per group. Data are expressed as means ± SEM. ^**^p < 0.01 vs non-diabetic; ^††^*p* < 0.01 vs type 1 diabetic *βGal*. ND, non-diabetic; T1DM, type 1 diabetic

Limb ischaemia was induced 13 weeks after gene delivery, with follow-up for 21 days. Compared with non-diabetic mice, diabetic mice showed delayed recovery of blood flow (Fig. 7a, b) and reduced reparative angiogenesis following induction of limb ischaemia (Fig. 7c–e). Pre-emptive gene therapy with Ad.h*NGF* normalised the reperfusion and capillary density of ischaemic muscles compared with diabetic control mice (Fig. 7a–e).

**Fig. 8:**
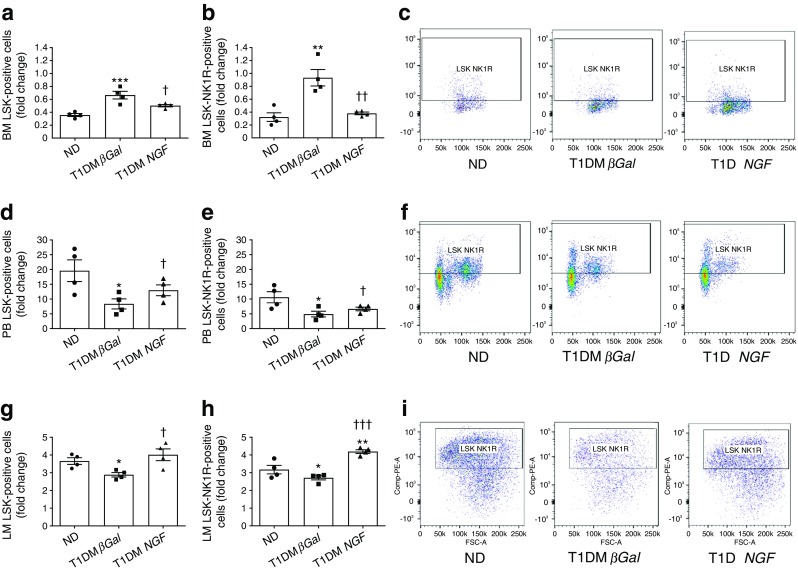
Systemic *NGF* therapy restores proper mobilisation of nociceptor-expressing cells in diabetic mice with limb ischaemia. (**a**–**c**) Bar graphs (**a**, **b**) and representative gating (**c**) from the flow cytometry analysis of bone marrow collected 3 days after limb ischaemia in non-diabetic and type 1 diabetic mice treated with Ad.*βGal* or Ad.h*NGF*. (**d**–**f**) Bar graphs (**d**, **e**) and representative gating (**f**) from the flow cytometry analysis of cells in peripheral blood collected 3 days after limb ischaemia. (**g**–**i**) Bar graphs (**g**, **h**) and representative gating (**i**) from the flow cytometry analysis of adductor limb muscles collected 3 days after limb ischaemia. Data are expressed as fold change in the number of positive cells vs pre-ischaemia (**a**, **b**, **d**, **e**) or contralateral limb muscle (**g**, **i**). *n* = 4 per group. Data are expressed as means ± SEM. ^*^p < 0.05, ^**^p < 0.01 and ^***^p < 0.001 vs non-diabetic mice; ^†^*p* < 0.05, ^††^*p* < 0.01 and ^†††^*p* < 0.001 vs type 1 diabetic *βGal* mice. BM, bone marrow; LM, limb muscle; ND, non-diabetic; PB, peripheral blood; T1DM, type 1 diabetic

**Fig. 9:**
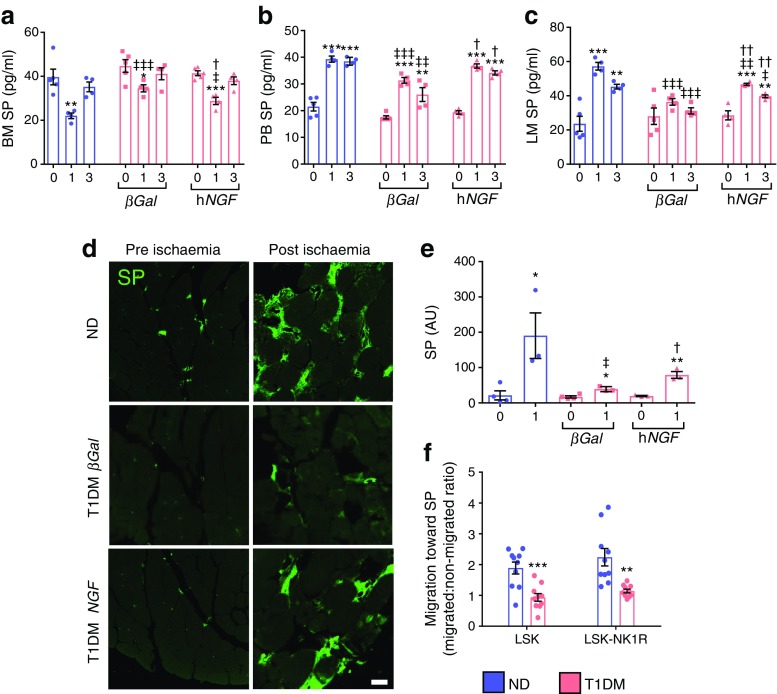
Systemic *NGF* therapy restores the substance P gradient in diabetic mice with limb ischaemia. (**a**–**c**) Bar graphs showing the results of ELISA measurements of substance P in bone marrow (**a**), peripheral blood (**b**) and limb muscles (**c**) before (day 0) and after (days 1 and 3) induction of limb ischaemia in non-diabetic and type 1 diabetic mice treated with Ad.*βGal* or Ad.h*NGF*. *n* = 5 per non-diabetic group 0 and 3 days; *n* = 4 per remaining groups. (**d**, **e**) Representative images (**d**) and bar graph (**e**) showing the expression of substance P in limb muscles before (day 0) and after limb ischaemia (day 1). Scale bar, 20 μm. *n* = 3 per group. ^*^p < 0.05, ^**^p < 0.01 and ^***^p < 0.01 vs pre-ischaemia; ^†^*p* < 0.05 and ^††^*p* < 0.01 vs type 1 diabetic mice treated with Ad.*βGal*; ^‡^*p* < 0.05, ^‡‡^*p* < 0.01 and ^‡‡‡^*p* < 0.001 vs non-diabetic mice. (**f**) Bar graph showing the in vitro migration capacity of LSK and LSK-NK1R bone marrow cells from non-diabetic and type 1 diabetic mice towards substance P. *n* = 10 per group; ^**^p < 0.01 and ^***^p < 0.001 vs non-diabetic mice. All data are expressed as means ± SEM. AU, arbitrary units; BM, bone marrow; LM, limb muscle; ND, non-diabetic; PB, peripheral blood; SP, substance P; T1DM, type 1 diabetic

We next investigated if gene therapy with *NGF* rescues progenitor cell mobilopathy in diabetic mice. Flow cytometric quantification of LSK-NK1R cells was performed in bone marrow, peripheral blood and ischaemic adductor muscles of non-diabetic and diabetic mice at 3 days post ischaemia. As seen before, both LSK and LSK-NK1R cells were reduced following limb ischaemia in the bone marrow (Fig. 8a–c) and increased in peripheral blood (Fig. 8d–f) and ischaemic muscles (Fig. 8g–i) of non-diabetic mice, with these effects being blunted by type 1 diabetes (Fig. 8a–i). Ad.h*NGF* therapy promoted the mobilisation and homing of LSK and LSK-NK1R cells (Fig. 8a–i). Measurement of substance P by ELISA showed a marked reduction in the neurokinin in bone marrow (Fig. 9a) and a concomitant increase in peripheral blood (Fig. 9b) and ischaemic muscles (Fig. 9c) of non-diabetic mice. This mobilising gradient was blunted in diabetic mice and restored to normal by Ad.h*NGF* therapy (Fig. 9a–c). We verified the expression and localisation of substance P in limb muscles by immunohistochemistry (Fig. 9d). In non-ischaemic skeletal muscle, substance P expression was localised at the level of the microvasculature. At 1 day post ischaemia, substance P expression was increased and became more diffuse in non-diabetic mice, but the effect was blunted by type 1 diabetes (Fig. 9d, e). Ad.h*NGF* therapy induced upregulation of substance P in ischaemic muscles of diabetic mice (Fig. 9d, e).

As unresponsiveness to chemoattractants is a key determinant of the defective release of bone-marrow-derived progenitor cells in individuals with diabetes [25–27], we also investigated whether NK1R-expressing cells of diabetic mice became insensitive to substance P stimulation. In line with this possibility, we found that the in vitro substance P-induced migration of bone marrow LSK-NK1R cells isolated from diabetic mice was remarkably reduced (Fig. 9f, *p* < 0.05 vs non-diabetic mice). Therefore, in the type 1 diabetes model used in these experiments, two mechanisms may fail following ischaemia: the ability to create a gradient of substance P between peripheral tissue and bone marrow; and the capacity of NK1R cells to respond to substance P-induced chemoattraction. The in vivo experiment indicates that prevention of neuropathy by NGF allows for proper activation of bone marrow cell mobilisation.

## Discussion

This study shows, for the first time, the presence of neuropathy involving NGF-dependent sensory neurons in bone marrow of type 1 diabetic mice. NGF supplementation prevents neuropathy, preserves the liberation of NK1R-expressing cells and benefits the recovery from ischaemia.

Previous work showed that physiological signalling through bone marrow adrenergic nerves regulates the circadian release of progenitor cells into the peripheral circulation [27, 28]. Moreover, we were the first to report that specialised sensory receptors for detecting noxious stimuli play key roles in tissue healing. Specifically, ischaemic pain potently stimulates the release of nociceptor-expressing pro-angiogenic progenitor cells in mice and humans [24]. Blockade of the sensory circuit by the opioid agonist morphine or by cardiac denervation abolished progenitor cell release and homing to ischaemic tissues [24]. Moreover, mouse bone marrow reconstitution with nociceptor-knockout progenitor cells resulted in delayed the recovery blood flow and reduced neovascularisation after ischaemia [24]. This led us to hypothesise that the pain associated with a heart attack or acute injury is playing two roles, that is, it is acting as a sensor alarm and a switch for healing circuits. Some diabetic people experience ‘silent’ heart attacks because of sensory neuropathy, which may potentially leave them without those useful defence mechanisms [29]. Accordingly, we demonstrated that individuals with neuropathic type 2 diabetes do not release nociceptor-expressing progenitor cells from bone marrow under ischaemia or following granulocyte-colony stimulating factor (G-CSF) stimulation [8].

Sensory neuropathy is not an exclusive feature of elderly people with type 2 diabetes. A recent investigation of the participant to the SEARCH for Diabetes in Youth Study (SEARCH) estimated that the prevalence of neuropathy was 7% in young people with type 1 diabetes and 22% in young people with type 2 diabetes, but the difference was abrogated after adjustment for factors such as diabetes duration, blood pressure and waist circumference [9] The authors concluded that the early manifestation of neuropathy is a cause of concern and suggest that early screening and better risk factor management are needed.

Our findings add another piece of evidence about the deleterious effect of diabetes on bone marrow anatomy and function [30–32]. We show, for the first time, the presence of nociceptive fibre rarefaction in the bone marrow of young mice with STZ-induced type 1 diabetes. Diabetic mice had a 2.3-fold reduction in the density of fibres expressing the pan-neuronal marker PGP9.5 and a 3.3-fold decrease in nociceptive fibres positive for substance P and NGF, suggesting a more severe impact on sensory nerves. Substance P-positive sensory fibres were detected in the bone and around capillaries in the marrow parenchyma. This perivascular location is in keeping with the notion that nerves can regulate cell mobilisation from the bone marrow by influencing vascular permeability [27]. The anatomical alteration of bone marrow sensory innervation was associated with a reduced liberation of LSK cells that express the substance P receptor NK1R on induction of limb ischaemia. The basal levels of LSK-NK1R-positive cells resident in limb muscles were very low, in the range of 1% of total isolated cells. It is well known that a small fraction of haematopoietic progenitor cells constantly circulate between the bone marrow and peripheral blood without any stimulation following a circadian rhythm [28]. It has also been proposed that several peripheral tissues, particularly peripheral fat, can constitute an extramedullary reservoir for functional haematopoietic stem and progenitor cells [33]. Our study suggests that the abundance of this resident population is not altered in the skeletal muscle of diabetic mice. On the other hand, the impact of type 1 diabetes on LSK-NK1R-positive cells homing on limb ischaemia was remarkable, with abolition of muscular colonisation observed in non-diabetic control animals. This defective homing may have contributed to the poor angiogenic and perfusion recovery seen in diabetic mice. In fact, LSK-NK1R-positive cells possess pro-angiogenic and healing capacities and their abrogation by genetic or pharmacological approaches results in defective reparative angiogenesis [24]. The defective recruitment could be attributed to the incapacity of peripheral and bone marrow sensory neurons to coordinate a proper gradient of substance P instrumental to the attraction of LSK-NK1R-positive cells, but also to an intrinsic lack of migratory capacity of these cells in response to chemoattractants, as observed in the in vitro assay of migration towards substance P.

The neurotrophic factor NGF supports neuronal survival and growth during prenatal development and maintains neuronal homeostasis postnatally through binding to TrkA [34]. In line with this, we observed that, in vivo, *NGF* therapy activated the TrkA signalling, as shown by the phosphorylation of rpS6 in the cell bodies of substance P-positive sensory neurons. Moreover, our in vitro findings corroborate that increased environmental levels of hNGF promote neurite outgrowth, prevent apoptosis and stimulate the canonical signalling pathway downstream to TrkA, namely phosphorylation of ERK1/2, p70S6K and CREB. On the other hand, the main function of p75, which is the preferred receptor for proNGF, remains elusive. It has been shown to promote cell survival either in association with TRK receptors or by itself [35, 36] and also to induce apoptotic cell death [37, 38]. Cell death induced by p75 reportedly requires c-JNK activation. In our investigation, the in vitro exposure of PC12 cells to high glucose slightly increased JNK phosphorylation. This effect of high glucose persisted following incubation of the same cells with the conditioned medium from Ad.h*NGF*-transduced fibroblasts, which was rich in mature NGF but not proNGF. On the other hand, the NGF-rich conditioned medium prevented the activation of apoptotic signalling induced by high glucose, as assessed by measurement of caspase 3/7 activity. Altogether, these data suggest that, in our experimental setting, induction of the survival signalling pathway, ERK1/2, p70S6K and CREB, rather than inhibition of p75/JNK, contributed to the improved survival of PC12 cells. Likewise, the protective effect of *NGF* gene therapy is highlighted by its ability to contrast the rarefaction of substance P-positive fibres in mouse bone marrow.

In the last decade, some studies have investigated the potential therapeutic benefit of NGF in neuropathic conditions. Some clinical trials have indicated that NGF is safe and effective as a treatment for preventing progression of diabetic or human immunodeficiency virus-associated peripheral neuropathies at the highest dose of 0.3 μg/kg two or three times per week [39, 40]. Another trial found no significant benefit in diabetic peripheral neuropathy at the dose of 0.1 μg/kg three times per week, thus suggesting a dose-related mechanism [39]. In line with this possibility, large improvements in pain intensity were observed following high-dose NGF [40]. Nevertheless, a controversy is emerging from preclinical and clinical trials regarding the potential for improvement or exacerbation of neuropathic pain with NGF therapy [41–43]. We did not perform behavioural tests in our study, and so the possibility that protection from sensory neuropathy is associated with neuropathic pain cannot be excluded.

We were the first to demonstrate that local supplementation of the NGF protein accelerates recovery from limb ischaemia by potentiation of skeletal muscle angiogenesis [15]. We have also shown that, in non-diabetic mice with myocardial infarction, systemic *NGF* gene therapy promotes detachment of progenitor cells from the endosteal niche through activation of metalloproteinase-9 [18]. Here, we provide novel evidence that pre-emptive treatment with *NGF* improves post-ischaemic blood flow recovery and restores proper neurokinin signalling instrumental to the release and homing of LSK-NK1R-positive cells. A direct effect of NGF on muscle neovascularisation cannot be excluded. However, at variance from our previous studies in which NGF was administered as a protein or gene at the time of the ischaemia [15–18], here we administered *NGF* therapy 13 weeks before the induction of ischaemia. Therefore, it is likely that the therapeutic effect is also attributable to preventive actions of NGF, one of which is protection against diabetes-induced bone marrow neuropathy. Although neuropathy does not directly cause vascular damage, the two conditions synergise in worsening diabetic foot complications.

In conclusion, we report that bone marrow neuropathy occurs early in the course of type 1 diabetes, resulting in alteration of pro-angiogenic cell capacity to migrate to sites of tissue injury. Pre-emptive gene therapy with *NGF* corrected this defect and improved the recovery from limb ischaemia. Although acute occlusion of the femoral artery may not reflect chronic vascular disease occurring in people with diabetes, our findings suggest that prophylactic treatment of sensory neuropathy by neurotrophic factor therapy could be a viable option for alleviating neurotrophic complications in type 1 diabetes.

## Electronic supplementary material

ESM (PDF 480 kb)

## Data Availability

The datasets used and/or analysed during the current study are available from the corresponding author on reasonable request.

## Abbreviations

Ad.*βGal*: Adenovirus carrying the β-galactosidase gene
Ad.h*NGF*: Adenovirus carrying the human *NGF* gene
CREB: cAMP response element binding protein
DRG: Dorsal root ganglia
ERK1/2: Extracellular signal-regulated kinases 1/2
GDNF: Glial-cell-line-derived neurotrophic factor
JNK: Jun N-terminal kinase
LSK: Lineage^−^ Sca-1^+^ and c-Kit^+^ cells
NGF: Nerve growth factor
NK1R: Neurokinin 1 receptor
P75^NTR^: p75 neurotrophin receptor
P-rpS6: Phospho-ribosomal protein S6
RET: Receptor for GDNF-family ligands
SAPK: Stress-activated protein kinase
STZ: Streptozotocin
TrkA: Neurotrophic tyrosine kinase receptor type 1
