# Supplementary material for: Nerve growth factor gene therapy improves bone marrow sensory innervation and nociceptor-mediated stem cell release in a mouse model of type 1 diabetes with limb ischaemia
Source: Diabetologia. 2019 Apr 24;62(7):1297–311. doi: 10.1007/s00125-019-4860-y (PMC6560027; doi:10.1007/s00125-019-4860-y)
Supplement: Supplementary file 1 — (PDF 480 kb) [file 125_2019_4860_MOESM1_ESM.pdf]

Nerve growth factor gene therapy improves bone marrow sensory innervation and nociceptor-mediated stem cell release in a mouse model of type 1 diabetes with limb ischaemia

ESM - Methods

Materials: unless otherwise stated, all reagents and chemicals have been purchased from Sigma-Aldrich, Gillingham, Dorset, UK.

Induction of diabetes and LI

The housing, care and procedures in all mice (*Mus musculus)* used in these studies was in accordance with and under license of the Animals (Scientific Procedures) Act 1986 (London, United Kingdom), and conform to the Guide for the Care and Use of Laboratory Animals published by the U.S. National Institutes of Health (Publication No. 85–23, revised 1996). The study received local institutional review board (University of Bristol, Bristol, United Kingdom) approval. The mice were housed in groups of 2-6 animals in an enriched environment within a biosecure unit, fed with EURodent Diet 22% (5LF5, LabDiet, St. Louis, MO, USA) and given water *ad libitum*. All surgery was performed using aseptic conditions under isoflurane anaesthesia, with buprenorphine analgesia.

Type 1 diabetes was induced in male 7-weeks old CD1 mice (Hsd:ICR(CD-1®), obtained from Harlan, Bicester, UK) by intraperitoneal injection of streptozotocin (STZ, 40 mg/kg body weight per day for 5 days) [1-3]. STZ-induced diabetic mice develop a form of neuropathy like that seen in type 1 diabetic patients [4-6]. Age-matched male CD1 mice injected with STZ-vehicle were used as non-diabetic (ND) controls.

Unilateral limb ischaemia (LI) was induced in mice under isoflurane anaesthesia, as described [7].

***In vivo* gene therapy and samples harvesting**

The experimental protocol is summarised in **ESM Fig. 2**. Adenoviral vectors carrying either the V5-tagged human *NGF* or *β-GAL* were produced as previously described [8]. The presence of the V5-tag allows easy tracking of the human protein produced by the Adenovirus. Two weeks after the first STZ injection, diabetic mice were injected with either Ad.*hNGF* or Ad.*βGal via* the tail vein (total dose of 1.5×10^9 viral particles in 100 µL). Age-matched nondiabetic mice were injected with Ad.*βGal and* used for reference. At sacrifice (16 weeks post gene transfer), bone marrow (BM) from tibias and femurs and spinal cord were collected for histological analyses.

LI ischaemia was induced as described above 13 week after gene delivery with follow-up for 21 days. At sacrifice (0, 1, 3, 7, 14, 21 days after LI), peripheral blood (PB) and bone marrow (BM) from tibias and femurs were collected for assessment of neuropeptide levels and flow cytometry analysis of cell antigenic profile. Hindlimb muscles (ischaemic and contralateral) were collected for assessment of vascular density.

An additional set of CD1 male mice (without diabetes or LI) were injected with Ad.*hNGF* or Ad.*βGal via* the tail vein (total dose of 1.5×10^9 viral particles in 100 µL).

Plasma was collected at sacrifice 3 days later for assessment of hNGF overexpression.

Immunohistochemistry and immunofluorescence microscopy

Samples harvesting and processing. Femoral bones were collected and cleaned from surrounding tissues, rinsed with phosphate-buffered saline (PBS), fixed with 4% w/v PFA for 24 hours at 4˚C and decalcified in 10% v/v formic acid for 48 hours at 4˚C. Then, after a brief post-fixation with 4% PFA for 4 hours at 4˚C, the bones were rinsed with PBS and processed for paraffin embedding. The same protocol has been applied to spinal cord samples. Hindlimb muscles were fixed with 4% w/v PFA for 16 hours at 4˚C and paraffin-embedded. Four µm-thick longitudinal sections were prepared for histological evaluation. For all antigens, heat-based antigen retrieval was performed by microwave (3 min x 3, 800W), either in sodium citrate buffer 0.01 mol/l, pH=6, or 0.05% v/v Tween-20 Tris/EDTA buffer, pH 9. For phospho-rpS6, antigen-retrieval was performed using a controlled-temperature water-bath (sodium citrate buffer 0.01mol/l, 98 C, 10 min).

Immunohistochemistry: For light microscopy assessment (visualised using diaminobenzidine (DAB) and alkaline phosphatase (AP)), endogenous peroxidase activity was blocked using H_2O_2 3% v/v. REAL EnVision/HRP, Rabbit/Mouse (ENV) (Dako, Santa Clara, CA, USA) was used for detecting the primary antibodies, in accordance with the protocols described in **ESM Table 1**. The reactions were revealed by a solution (1:50) of REAL DAB+ Chromogen and REAL Substrate Buffer (Dako, Santa Clara, CA, US). Nuclei were stained with Mayer's haematoxylin. All staining steps were performed at 15-25°C.

Immunofluorescence: Sections were blocked with 10% v/v non-immune normal goat serum (Cell Signalling Technology, London, UK). Sections were then incubated with primary and secondary antibodies as shown in **ESM Table 1.** Secondary antibodies were either conjugated directly with AlexaFluor or with streptavidin followed by an AlexaFluor step. All antibodies have been diluted with 5% v/v non-immune normal goat serum. Secondary antibodies were purchased from LifeTechnologies (Thermo Fisher Scientific, Waltham, MA, USA). Nuclei were stained with DAPI (1:1000 v/v in PBS) for 2 min, and slides were mounted with Fluoromount-G (Thermo Fisher Scientific).

Quantification: Bone marrow (BM) neuronal fibres were expressed as number/mm^2. NGF and substance P (SP) positive staining in BM and muscles was expressed as the percentage of the total area assessed. Capillary and arteriole densities were expressed as number of vascular profiles/mm^2. A minimum of 10-15 images taken randomly with a 100-200x total optical magnification was analysed.

Plasma isolation

PB was collected with EDTA as an anticoagulant and kept on ice until processed. Samples were centrifuged for 15 min at 1000 x g at 4°C. Plasma was aliquoted and stored at -80°C for subsequent analyses using ELISA assays.

ELISA assays

ELISA assays for hNGF (Cloud-Clone Corp., Katy, TX, USA) and pro-hNGF (CUSABIO, Houston, TX, USA) were performed according to manufacturer instructions on plasma collected from animals 3 days post-gene transfer to assess the levels of the circulating proteins. All samples were assayed in duplicates. This was used also to confirm that the mature hNGF was the only form of the recombinant protein.

ELISA for SP (Cayman Chemical Company, Ann Arbor, Michigan, USA) was employed to detect SP levels in mice BM, plasma, and ischaemic adductor muscles. Briefly, samples were diluted in 2M acetic acid, fractionated with C-18 reverse-phase single-use columns (Cayman Chemical Company), and concentrated under vacuum. Quantification of SP was performed by measuring the changes in the absorbance of nitrobenzoic acid at 412 nm.

Competitive assay to check specificity of the anti-NGF antibody

A peptide competition assay was performed to rule out non-specific binding of the anti-NGF antibody to other proteins in the mouse tissues. The NGF peptide was purchased from Abcam (Cambridge, UK) (Ab154297). This peptide was recommended by the vendor to block the anti-NGF antibody Ab52918. According to manufacture instructions, the antibody was incubated with five times excess blocking peptide by weight in blocking solution (PBS + 1% w/v BSA), for 30 min at RT, with gentle agitation. A tube with the antibody but without peptide served as control. The staining of the BM tissue was performed following the normal protocol using the blocked and control antibody on two different slices of the same samples.

Culture and characterisation of PC12 cells

PC12 cells were cultured in DMEM containing 1g/l Glucose + Glutamine, 10% v/v FBS and pen/strep and passaged at confluence every 3 days. For phenotypic characterisation, cells were fixed with 4% w/v PFA for 15 min at 15-25°C and permeabilised using 0.1% TritonX for 5 min as required. After blocking with 10% v/v normal goat serum (Cell Signalling Technology), antibodies were incubated for 16 h at 4°C (as reported in **ESM Table 1)**. Alexa488-conjugated secondary antibodies raised in goat were used to visualise the staining, and the nuclei counter-stained using DAPI. Cells were analysed at a x200 magnification.

Rat fibroblasts transduction and collection of conditioned media

Primary rat fibroblasts were cultured in DMEM containing 1g/l glucose with glutamine, 10% v/v FBS and pen/strep, and passaged at confluency (~3 days). Cultures that were 80% confluent were transduced with either Ad.*hNGF* or Ad.*βGal* at a total dose of 5 × 10^7 viral particles/ml. Cells not transduced served as control (NV, no virus). After 48h, the conditioned media (CM) was centrifuged for 10 min at 1000 x g at 4°C to remove floating cells/debris and frozen for subsequent studies with PC12 cells. An aliquot of medium was used to check the expression of the human NGF using western blotting.

A further aliquot was used to measure the amount of NGF and proNGF using ELISA assays.

Biological assays on PC12 cells exposed to the CM from NGF-transduced fibroblasts

PC12 cells were maintained under basal (BG, 5 mmol/l) or high (HG, 30mmol/l) D-Glucose. Mannitol (25mmol/l) was used as osmotic control when required.
For *signalling experiments*, PC12 pre-conditioned for 48h with BG or HG media were exposed to the fibroblasts CM (NV, hNGF, βGAL) for 30 min and total cell extracts were collected for Western Blotting.
For *Caspase 3/7 apoptosis assay*, PC12 were incubated for 48h with the fibroblasts CM in the presence of either BG or HG or Mannitol. At the end of this incubation time, the Caspase Glo 3/7 Assay (Promega, Madison, Wisconsin, USA) was used to measure caspase activity. This was quantified as relative luminescence units (RLU) and is expressed as fold change compared with the BG-NV group, using 4 replicates per group.
For *neuronal differentiation and neurites outgrowth experiments*, low density PC12 were incubated for 72h with the fibroblasts CM in the presence of either BG or HG. At the end of the protocol, images were snapped using a x 200 magnification. The total neurites length per cell was measured as an index of neurites outgrowth (N=50 cells randomly measured in 3-4 different optical fields), while cells presenting at least one axon longer than the cell body were considered as differentiated “neuron-like” cells and expressed as a percentage of the total number of cell (per each group, N=250 to 300 cells analysed in 4 different optical fields).
For all experiments, fibroblasts CM was diluted 1:10 with fresh culture media, with appropriate adjustment for the final level of glucose or mannitol. The final hNGF concentration given to the PC12 cells was 540 pg/ml, as measured by ELISA assay.

Proteins extraction and western blotting

Total proteins from cells were obtained using RIPA lysis buffer supplemented with protease and phosphatase inhibitors cocktails. In addition, CM from cells was collected for measuring secreted proteins. This was centrifuged for 10 min at 1000 x g at 4°C to remove floating cells/debris and supplemented with Laemmli loading buffer. The protein concentration in cell extracts was determined using the BCA Protein Assay Kit (Thermo Fisher Scientific). Equal amounts of protein samples (10-15μg) were prepared in Laemmli loading buffer, incubated for 8 min at 98°C, resolved on 8-12% SDS-PAGE according to the proteins MW and transferred onto 0.2 μm PVDF membranes (Bio-Rad, Hercules, California, USA). Membranes were blocked using 5% w/v non-fat dried milk in Tris-buffered saline (TBS) supplemented with 0.05% v/v Tween-20 (TBS-T) for 2 h at 15-25°C. Primary antibodies - listed in **ESM Table 1** - were incubated for 16h at 4°C using 5% w/v BSA in TBS-T. Secondary antibodies - listed in **ESM Table 1** - were incubated for 1h at 15-25C using 5% w/v non-fat dried milk in TBS-T. β-tubulin was used as loading control. Membrane development was performed by an enhanced chemiluminescence-based detection method (ECL™

Prime Western Blotting Detection Reagent, GE Healthcare, Chicago, Illinois, USA) in a ChemiDoc-MP system (Bio-Rad). Proteins with similar molecular weight were assessed on different gels. When necessary, no more than 2 stripping procedures were performed in the same membrane (RestoreTM Plus Western Blot Stripping Buffer, Thermo Scientific). All samples to be compared were loaded on the same gel for densitometry analysis. Data were analysed using the free ImageJ software to determine optical density (OD) of the bands. The OD readings of phosphorylated proteins were expressed as a ratio relative to non-phosphorylated protein (N=1 replicate). Final data are showed as the fold change of the BG-NV group (considered to be the basal control).

Flow cytometry

Analyses of progenitor cells in PB, BM and adductor muscles of mice were conducted using multicolour flow cytometry.

Cell collection. Freshly harvested cells were washed with ice-cold Hank's balanced salt solution containing 0.5% w/v BSA and 0.02% w/v sodium azide. Adductor muscles were harvested, minced with fine scissors and placed into a digest solution of Collagenase A 100 µg/µl (Roche, Basel, Switzerland), Dispase II (1X) 2.4 U/ml (Roche), DNase I 10 mg/ml (Roche), BSA 10% w/v, $CaCl_2$ 50 mmol/l, $MgCl_2$ 1 mol/l in PBS at 37°C for 1 h, triturated through 40µm diameter nylon mesh and washed with 0.2% w/v BSA + 0.1% w/v DNAse I in PBS.

Analysis: Cells were fixed and permeabilised using a fix/perm solution (BD, Franklin Lakes, New Jersey, USA) followed by blocking with anti-mouse CD16/32 (eBioscience, Thermo Scientific, Waltham, Massachusetts, USA) in PBS with 1% w/v BSA for 15 min at 4˚C. Cells were then incubated with lineage and stem cells/progenitor cell antibodies: anti-Lineage Mixture (mouse CD3e, CD11b, CD45R, Ly-6C/G, TER119, Invitrogen, carlsbad, California, USA) and anti-Ly6-A/E (Sca-1), anti-Cd117 (c-Kit) (both from eBioscience), and rabbit anti-Neurokinin 1 Receptor (NK1R) (Novus Biologicals, Centennial, Colorado, USA) for 30 min at 4˚C. The concentration of primary antibodies is reported in **ESM Table 1**. Cells were then washed, treated with Goat Anti-Rabbit PE secondary antibodies for 30 min at 4˚C, and finally analysed using a FACS Canto II equipped with FACS Diva software (BD).

BM cells migration

To verify the effect of type 1 diabetes on the sensitivity of BM cells to SP, we performed *in vitro* migration assays using 6 well-plates transwell cell culture inserts equipped with 3-5µm pore size filters (Corning, New York, USA). Freshly-isolated BM cells were plated onto the upper compartment ($2.5x10^6$, cells/well) and 100 mmol/l SP was added to the lower compartment in DMEM and 0.5% w/v foetal bovine serum. Cells were allowed to migrate overnight in a humidified atmosphere (37°C, 5% CO_2). Cells from the upper (non-migrated cells) and lower compartments (migrated cells) were collected and processed for flow cytometry analysis with the use of AccuCheck counting beads (Invitrogen) for absolute and reproducible quantification of cell numbers. Enrichment of antigenically-defined cell populations was expressed as the

ratio of cells in lower and upper chambers and then normalised to control (cells treated with vehicle).

Statistical analysis

The D'Agostino & Pearson omnibus normality test was applied to check for normal distribution of data. Normally-distributed continuous variables were expressed as mean ± SEM and compared between groups using the unpaired Student *t*-test. When continuous variables did not follow a normal distribution, values were expressed as median (interquartile range IQR) and compared with the Wilcoxon-Mann-Whitney test. Categorical variables were compared using the chi-squared or the Fisher's exact test. All reported *p*-values are two-sided. A *p*-value <0.05 was considered statistically significant. Statistical analyses were performed with STATA 11 software (StataCorp. 2009. Stata Statistical Software: Release 11. College Station, TX: StataCorp LP) or GraphPad Prism software, Version 6.01.

ESM Table 1. List of antibodies used in the experimental procedures

Antibody	Manufacturer	Catalogue number	IHC/ICC dilution (v/v)	FACS dilution (v/v)	WB dilution (v/v)	Detection System	IHC/ICC dilution (v/v)	FACS/WB dilution (v/v)
**Rabbit anti-PGP 9.5**	Millipore	AB5925	1:500			Peroxidase/DAB or TSA plus Fluorescein kit (NEL741E001KT)	1:50	
**Rabbit anti-Substance P**	Immunostar	22064	1:1500			Peroxidase/DAB or TSA plus Fluorescein kit (NEL741E001KT)	1:50	
**Mouse anti-Substance P**	Abcam	Ab14184	1:100			Goat anti-mouse Alexa Fluor 546	1:200	
**Rabbit anti-NGF**	Abcam	Ab6199	1:500			Peroxidase/DAB	-	
**Rabbit anti-NGF**	Abcam	Ab52918	1:250			Goat anti-rabbit Alexa Fluor 488	1:200	
**Rat anti-mouse CD31 (MEC13.3)**	BD Pharmingen	557355	1:100			Goat anti-rat Alexa Fluor 568	1:300	
**Isolectin B4 - Biotin**	Invitrogen	121414	1:100			Streptavidin Alexa Fluor 488	1:200	
**Mouse anti-CD3e, CD11b, CD45R, Ly-6C/G, TER119 (Lineage cocktail) Alexa Fluor 488**	Invitrogen	MLM20		1:100				
**Rat anti-CD117 APC-eFluor 780**	eBioscience Invitrogen	47-1171-82		1-400				
**Rat anti-mouse Ly-6A/E (Sca-1) APC**	eBioscience Invitrogen	17-5981-83		1:400				
**Rabbit anti-NK1 receptor**	Novus Biologicals	NB300-119B	1:100			Goat anti-rabbit PE		1:200
**Rabbit anti-p75**	Millipore	AB1554	1:200			Goat anti-rabbit Alexa Fluor 488	1:200	
**Rabbit anti-RET**	Abcam	Ab134100	1:150			Goat anti-rabbit Alexa Fluor 488	1:200	
**Rabbit anti-TRKA**	Abcam	Ab76291	1:50		1:500	Goat anti-rabbit Alexa Fluor 488<br>Anti-rabbit IgG HRP	1:200	1:5000
**Mouse anti-V5 Tag**	Thermo Fisher Scientific	R960-25	1:150		1:1000	Goat anti-mouse Alexa Fluor 546<br>Anti-mouse IgG HRP	1:200	1:5000
**Mouse anti-Substance P**	Abcam	Ab14184	1:100			Goat anti-mouse Alexa Fluor 546	1:200	
**Phospho-S6 Ribosomal Protein (Ser235/236)**	Cell Signaling	4858	1:300			Goat anti-rabbit Alexa Fluor 488	1:200	
**Phosphor-TRKA (Tyr490)**	Cell Signaling	9141			1:500	Anti-rabbit IgG HRP		1:5000
**AKT (pan) (40D4)**	Cell Signaling	2920			1:1000	Anti-mouse IgG HRP		1:5000
**Phospho-AKT (Ser473)**	Cell Signaling	9271			1:1000	Anti-rabbit IgG HRP		1:5000
**Phospho-CREB (Ser133)**	Cell Signaling	9191			1:1000	Anti-rabbit IgG HRP		1:5000
**CREB (86B10)**	Cell Signaling	9104			1:1000	Anti-mouse IgG HRP		1:5000
**Phospho-p44/42 MAPK (ERK1/2) (Thr202/Tyr204)**	Cell Signaling	4370			1:1000	Anti-Rabbit IgG HRP		1:5000
**p44/42 MAPK (ERK1/2) (137F5)**	Cell Signaling	4695			1:1000	Anti-Rabbit IgG HRP		1:5000
**Phospho-p70 S6 Kinase (Thr421/Ser424)**	Cell Signaling	9204			1:1000	Anti-Rabbit IgG HRP		1:5000
**p70 S6 Kinase (49D7)**	Cell Signaling	2708			1:1000	Anti-Rabbit IgG HRP		1:5000
**Phospho-SAPK/JNK (Thr183/Tyr185) (81E11)**	Cell Signaling	4668			1:1000	Anti-Rabbit IgG HRP		1:5000
**SAPK/JNK**	Cell Signaling	9252			1:1000	Anti-Rabbit IgG HRP		1:5000
**β-Tubulin**	Cell Signaling	86298			1:5000	Anti-mouse IgG HRP		1:5000

Antibodies validation: whenever possible, antibodies were previously validated by our team using positive or negative control tissues/cells. All antibodies used in this study have been previously used by other Investigators, and references are available either from the company or in online databases such as PubMed. The companies are often providing information on how antibodies specificity was tested.

ESM Figure 1

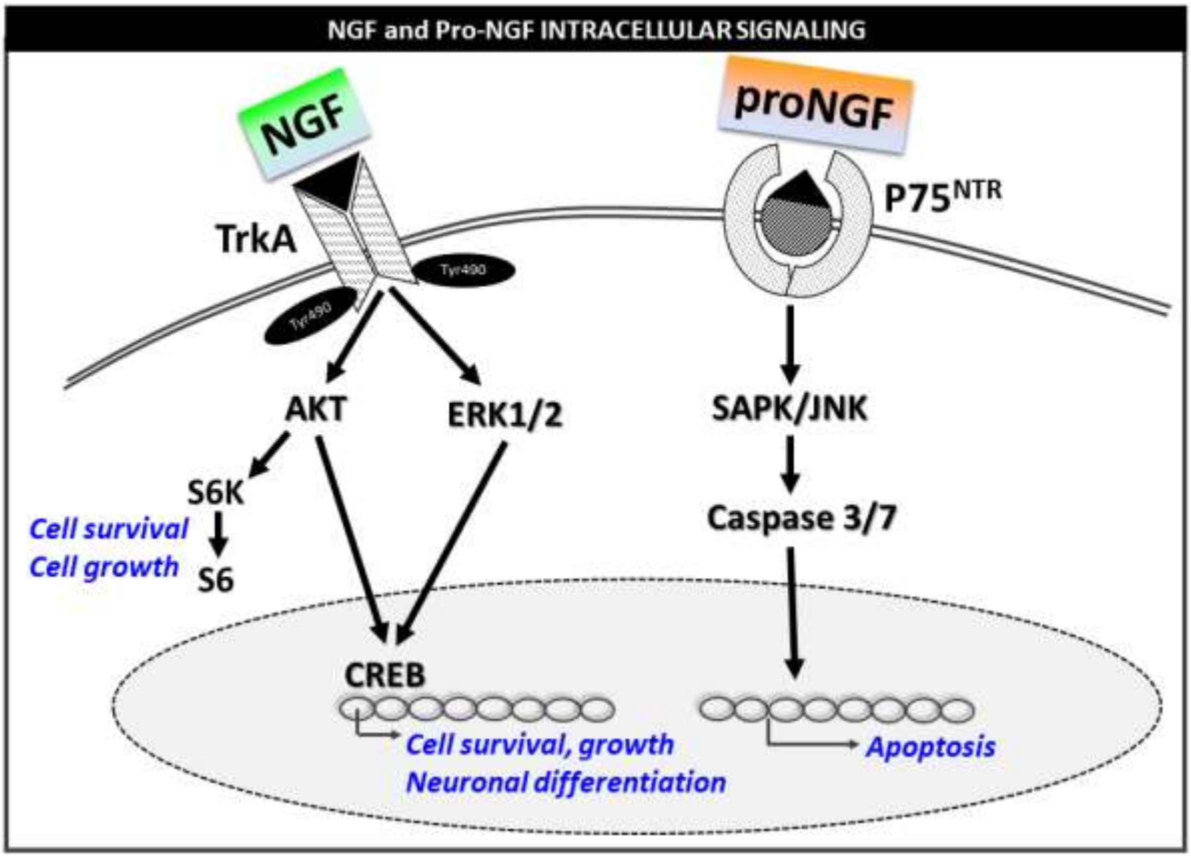

Schematic cartoon showing signalling pathways associated with NGF receptors. NGF binds with high affinity the TrkA receptor that promotes cell survival and neural differentiation. At the opposite, proNGF binds with high affinity the p75^{NTR} receptor, associated with cell death.

ESM Figure 2

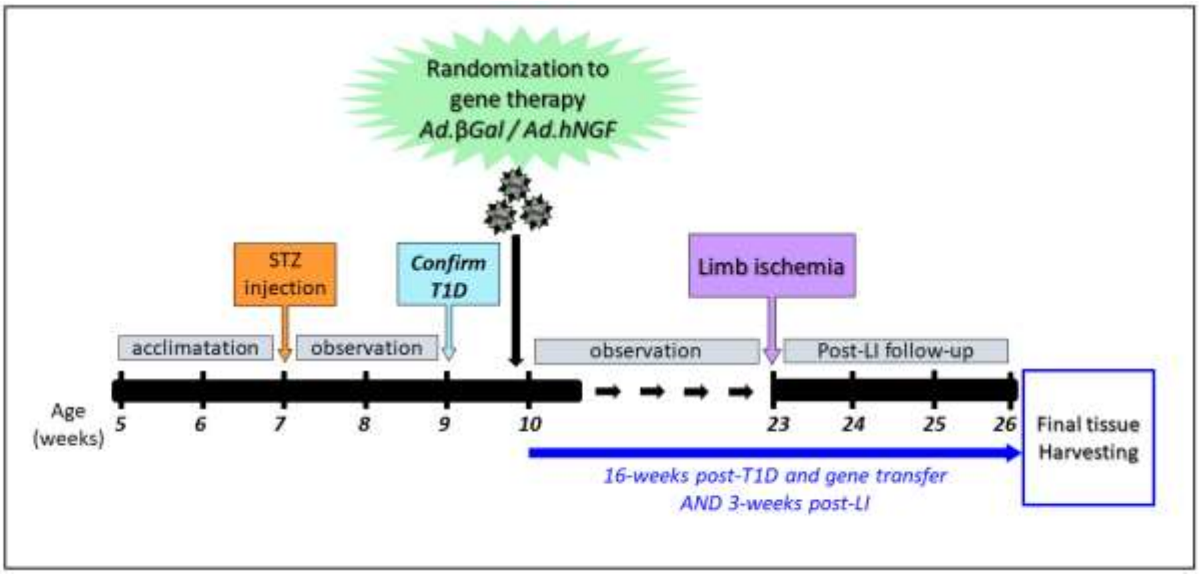

Schematic of the *in vivo* study experimental timeline.

ESM Figure 3

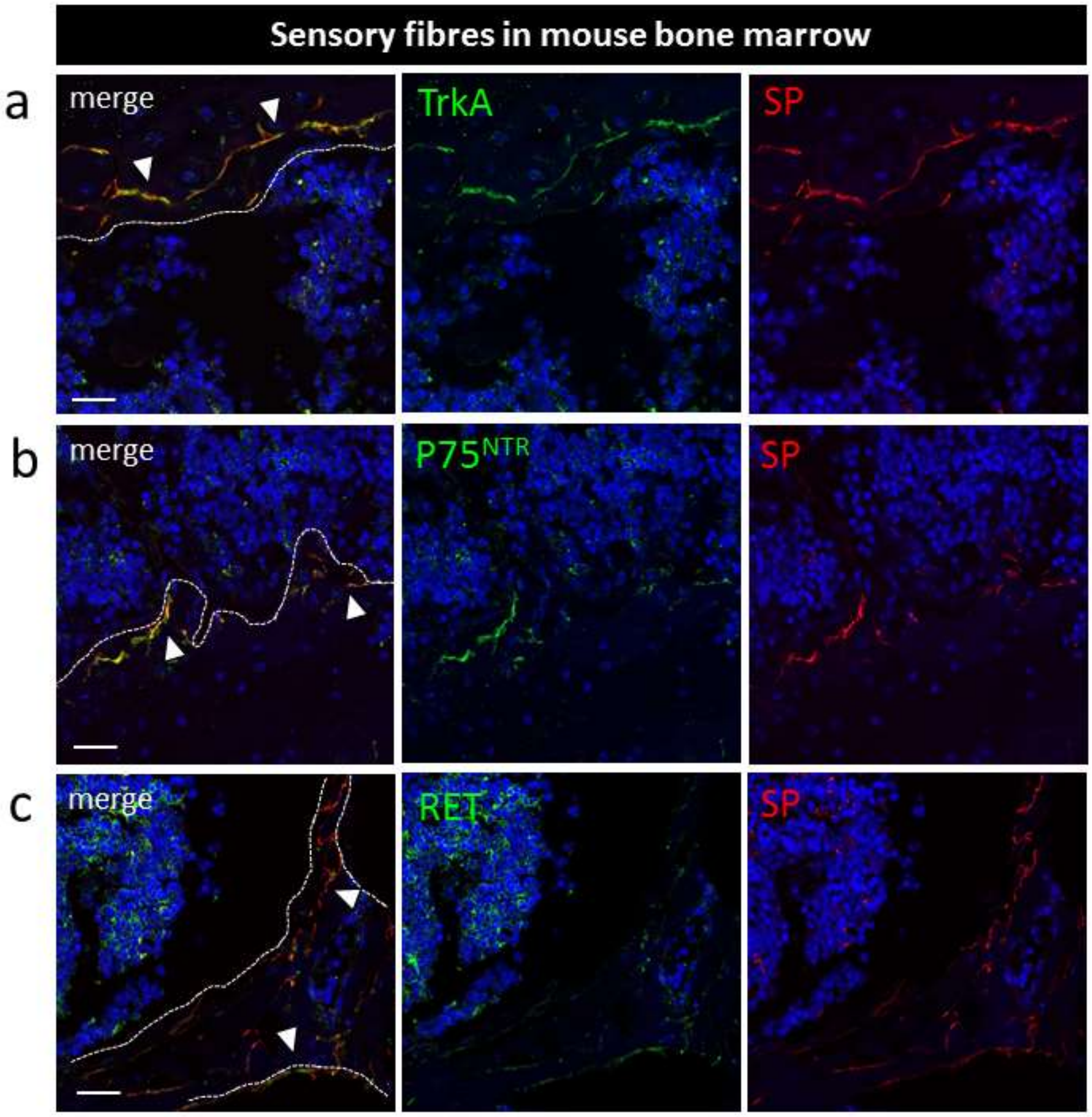

Sensory fibres in mouse bone marrow express neurotrophins receptors. (a-c) Sensory fibres in mouse bone marrow (BM) were identified by staining for substance P (SP, red fluorescence). Co-staining for TrkA **(a)**, p75^{NTR} **(b),** and RET **(c)** receptors, all in green fluorescence, demonstrated sensory fibres innervating the BM are potentially responsive to neurotrophins stimuli. DAPI (blue) identifies nuclei. Scale bar, 25 µm. Bone is identified by a dashed line.

ESM Figure 4

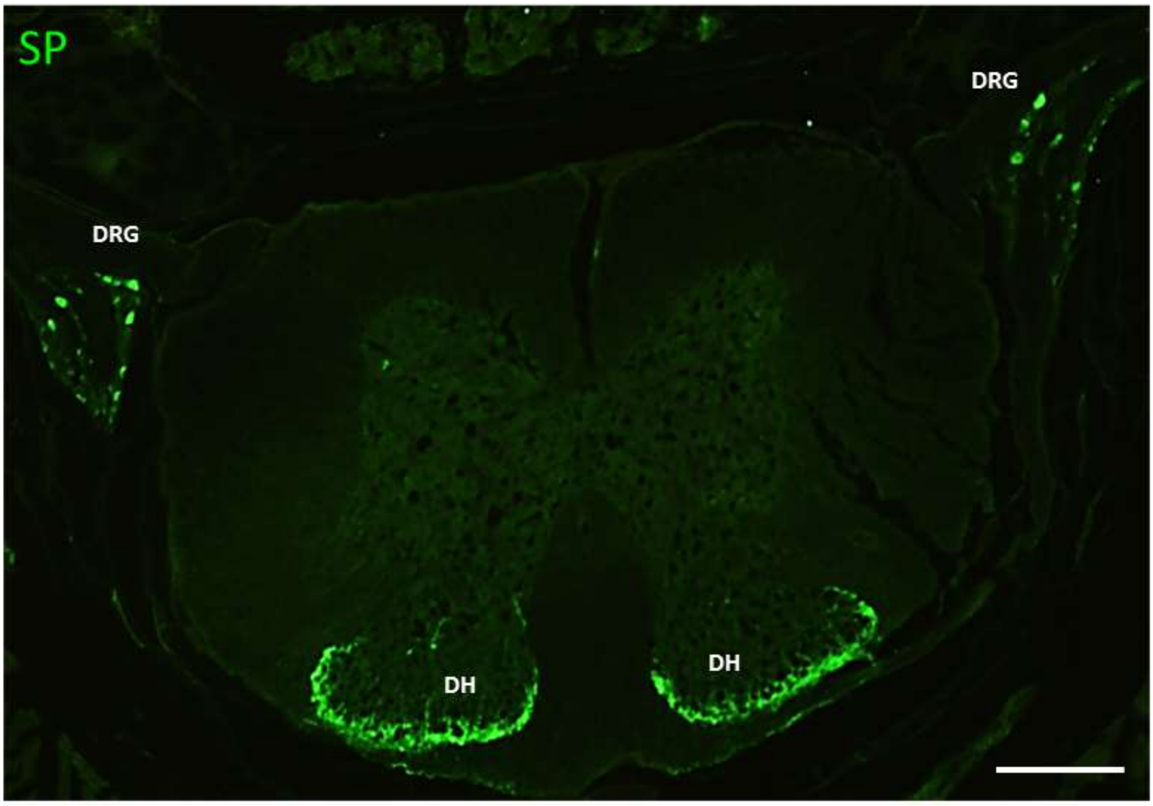

Dorsal root ganglia express substance P. Immunofluorescence staining for substance P (green) in the spinal cord. Dorsal root ganglia (DRG) and dorsal horns (DH) are positive to SP. Scale bar, 10 µm.

ESM Figure 5

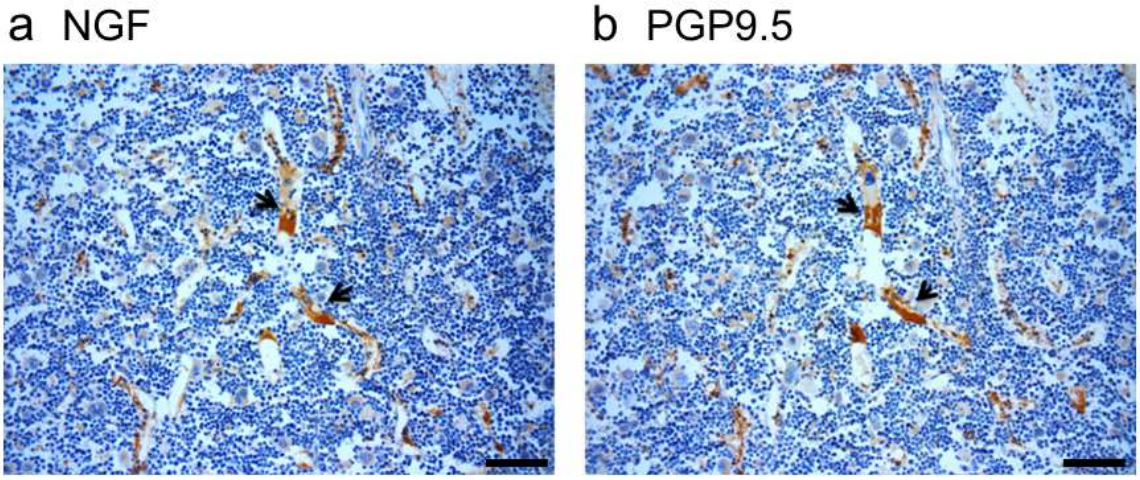

Expression of NGF in PGP9.5 neuronal fibres. Immunohistochemistry staining for NGF **(a)** and PGP9.5 **(b)** on two consecutive slices of bone marrow indicates fibres co-express the 2 markers. Scale bar, 50 µm.

ESM Figure 6

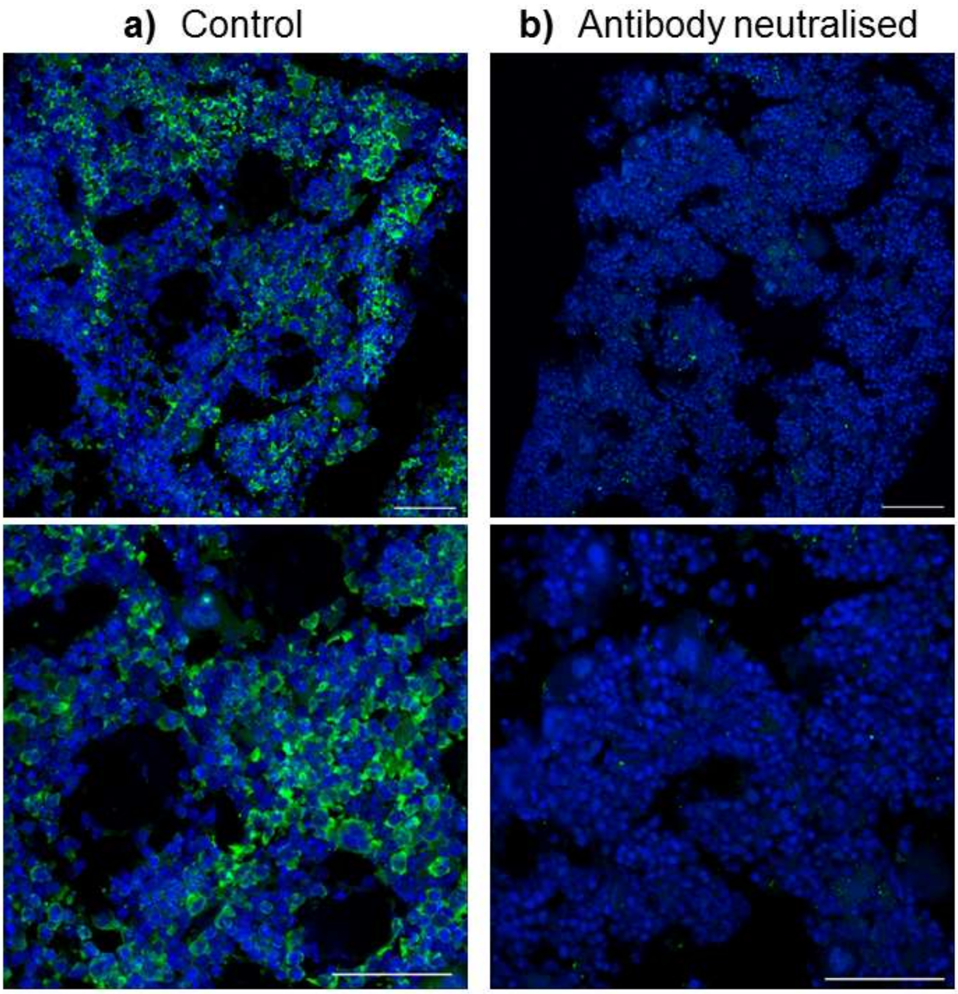

Competitive assay with NGF blocking peptide to test anti-NGF antibody (ab52918) specificity. a) control antibody; **b)** antibody pre-incubated with the NGF peptide. The neutralisation of the antibody prevented binding to the tissue. Images are provided at different magnification. Scale bar is 50 μm.

ESM Figure 7

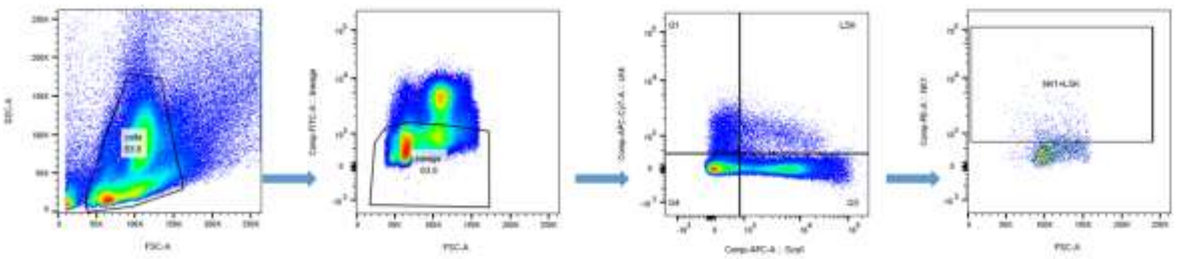

Flow cytometry strategy. Illustration of the gating strategy in flow cytometry analyses of LSK cells.

References

[1] Emanueli C, Graiani G, Salis MB, Gadau S, Desortes E, Madeddu P (2004) Prophylactic gene therapy with human tissue kallikrein ameliorates limb ischemia recovery in type 1 diabetic mice. Diabetes 53: 1096-1103

[2] Emanueli C, Salis MB, Pinna A, et al. (2002) Prevention of diabetes-induced microangiopathy by human tissue kallikrein gene transfer. Circulation 106: 993-999

[3] Graiani G, Emanueli C, Desortes E, et al. (2004) Nerve growth factor promotes reparative angiogenesis and inhibits endothelial apoptosis in cutaneous wounds of Type 1 diabetic mice. Diabetologia 47: 1047-1054

[4] Kusano KF, Allendoerfer KL, Munger W, et al. (2004) Sonic hedgehog induces arteriogenesis in diabetic vasa nervorum and restores function in diabetic neuropathy. Arteriosclerosis, thrombosis, and vascular biology 24: 2102-2107

[5] Kim H, Park JS, Choi YJ, et al. (2009) Bone marrow mononuclear cells have neurovascular tropism and improve diabetic neuropathy. Stem cells (Dayton, Ohio) 27: 1686-1696

[6] Anitha M, Gondha C, Sutliff R, et al. (2006) GDNF rescues hyperglycemia-induced diabetic enteric neuropathy through activation of the PI3K/Akt pathway. The Journal of clinical investigation 116: 344-356

[7] Madeddu P, Emanueli C, Spillmann F, et al. (2006) Murine models of myocardial and limb ischemia: diagnostic end-points and relevance to clinical problems. Vascular pharmacology 45: 281-301

[8] Meloni M, Cesselli D, Caporali A, et al. (2015) Cardiac Nerve Growth Factor Overexpression Induces Bone Marrow-derived Progenitor Cells Mobilization and Homing to the Infarcted Heart. Mol Ther 23: 1854-1866
